# Tunneling nanotubes and periodontal regeneration: a novel perspective on stem cell–immune cell interactions and osteoimmunity

**DOI:** 10.3389/fdmed.2026.1922083

**Published:** 2026-10-30

**Authors:** Deepa Ponnaiyan, Jaideep Mahendra, Gauri Patil, Muskan Bedi, Ganesh Shetty, Sakshi Belokar

**Affiliations:** 1 Department of Periodontics, Sri Ramaswamy Memorial Dental College Ramapuram, Chennai, Tamil Nadu, India; 2 Department of Periodontics, Meenakshi Ammal Dental College and Hospital, Meenakshi Academy of Higher Education and Research, Chennai, Tamil Nadu, India; 3 Department of Medicine, Sri Ramachandra Medical College and Research Institute, Sri Ramachandra University, Porur, Chennai, Tamil Nadu, India; 4 Dental and Orthodontic Clinic, Bangalore, India; 5 Sharad Pawar Dental College and Hospital, Wardha, Maharastra, India

**Keywords:** immune cells, intercellular communication, osteoimmunity, periodontal ligament stem cells, periodontal regeneration, tunneling nanotubes

## Abstract

The periodontal ligament (PDL) is a dynamic connective tissue essential for tooth support, homeostasis, and regeneration. Periodontal ligament stem cells (PDLSCs), located within the PDL, exhibit mesenchymal stem cell characteristics and represent a potential cell source for periodontal regeneration. Interactions between PDLSCs and immune cells are mediated by soluble factors, extracellular vesicles, and direct cell-to-cell contact. Tunneling nanotubes (TNTs) have emerged as specialized actin-based membranous structures that can establish intercellular connections and facilitate the transfer of mitochondria, proteins, nucleic acids, and other cellular components. TNT-associated communication has been implicated in cellular survival, inflammatory responses, and tissue repair in several experimental systems. However, the existence and functional significance of TNT-mediated communication between PDLSCs and immune cells in periodontal tissues remain insufficiently characterized. Findings from stem cell, immunological, and bone biology research provide a rationale for investigating whether TNT-associated communication influences periodontal osteoimmunity. Nevertheless, its effects on immune-cell function, osteoclastogenesis, and periodontal regeneration may depend on the participating cell types, transferred cargo, and inflammatory and metabolic conditions. This mini-review summarizes current knowledge of TNT biology, examines evidence relevant to PDLSC–immune cell interactions and osteoimmunity, and identifies knowledge gaps and research priorities for determining whether TNT modulation has therapeutic relevance to periodontal regeneration.

## 1 Introduction

Periodontitis is a chronic inflammatory osteoimmune disease which leads to the destruction of tooth supporting structure such as alveolar bone, periodontal ligament (PDL), cementum and gingiva (1). Although the disease is initiated by microbial dysbiosis, the majority of periodontal destruction is caused by the host innate and adaptive immune responses and altered osteoimmune homeostasis, which lead to the release of various pro-inflammatory cytokines, prostaglandins, and matrix metalloproteinases (MMPs), ultimately disrupting periodontal homeostasis (2, 3). The bidirectional crosstalk between skeletal and immune systems is termed osteoimmunity and plays an important role in the progression of periodontal disease (4). Chronic inflammation promotes osteoclast differentiation by disrupting the balance of the receptor activator of nuclear factor-*κ*B (RANK)/ receptor activator of nuclear factor-*κ*B ligand (RANKL)/osteoprotegerin (OPG) signaling axis, leading to alveolar bone resorption and impaired periodontal regeneration (5, 6).

The PDL is a specialized connective tissue which is located between the root cementum and the alveolar bone that provides support to the tooth while maintaining periodontal homeostasis and tissue repair. Among its resident cell populations, periodontal ligament stem cells (PDLSCs) are mesenchymal stem cells with a high self-renewal capacity, regenerative and multilineage differentiation potential. They can differentiate into osteoblasts, cementoblasts, fibroblasts and other mesenchymal lineages, thereby contributing to the regeneration of alveolar bone, cementum and the PDL (7). In addition to their capacity for regeneration, PDLSCs have strong immunomodulatory qualities and are essential for preserving the periodontal immune microenvironment (8, 9). The secretion of cytokines, chemokines, growth factors, extracellular vesicles and direct cell-to-cell contact are some of the mechanisms that mediate this bidirectional crosstalk (9, 10). These interactions suppress excessive inflammation and promotes tissue repair and provides suitable microenvironment for periodontal regeneration (8, 9). Existing evidence suggests that conventional communication pathways alone may not fully explain the direct interactions between PDLSCs and immune cells, prompting investigation into alternative mechanisms of intercellular communication.

Recently, tunneling nanotubes (TNTs) have emerged as a novel mechanism of direct intercellular communication. TNTs are thin actin-based membranes that create direct cytoplasmic connections between cells, allowing the transmission of mitochondria, proteins, nucleic acids and other bioactive compounds, in contrast to soluble mediators, extracellular vesicles or traditional cell-to-cell contact (11, 12). TNTs are linked to immune regulation and stem cell biology, but little is known about how they mediate communication between PDLSCs and immune cells in the periodontal milieu. Increasing evidence from stem cell biology, immunology, neuroscience, and bone biology has demonstrated that TNT-mediated communication contributes to cellular survival, immunomodulation and regenerative processes (13). Nevertheless, the existence and functional significance of TNTs within the periodontal microenvironment remain largely unexplored.

Given the central role of PDLSC–immune cell interactions in maintaining osteoimmune homeostasis and promoting periodontal regeneration, investigating whether TNTs mediate communication between these cells may provide new insights into inflammation, bone remodeling, and periodontal tissue repair (8–10). Elucidating these processes may also identify TNT-mediated signaling as a potential therapeutic target for enhancing periodontal regeneration. This mini-review summarizes current knowledge of TNT biology and discusses their emerging role in stem cell–immune cell communication, with particular emphasis on their potential contribution to periodontal regeneration and bone remodeling. It also highlights current knowledge gaps and future research priorities for translating TNT-based mechanisms into next-generation periodontal regenerative therapies (14).

## 2 Tunneling nanotubes: structure, formation and biological functions

### 2.1 Discovery and structural characteristics of TNTs

Tunneling nanotubes (TNTs) are long, thin actin-based membranous structures that connect distant cells through direct cytoplasmic continuity, allowing the selective transfer of membrane-bound organelles and intracellular cargo. Unlike gap junctions or extracellular vesicle-mediated communication, TNTs provide a direct bridge for long distance cell-to-cell communication (11, 12). TNTs are primarily composed of filamentous actin (F-actin), although thicker TNTs in certain cell types may also contain microtubules. TNTs were discovered by Amin Rustom and colleagues in 2004, while performing an experiment on cultured rat pheochromocytoma (PC12) cells and normal rat kidney cells. Their diameter generally ranges from 50 to 2000nm and their length may extend from several micrometers to over 100 μm, depending on the cell type (13) (Figure 1).

**Figure 1:**
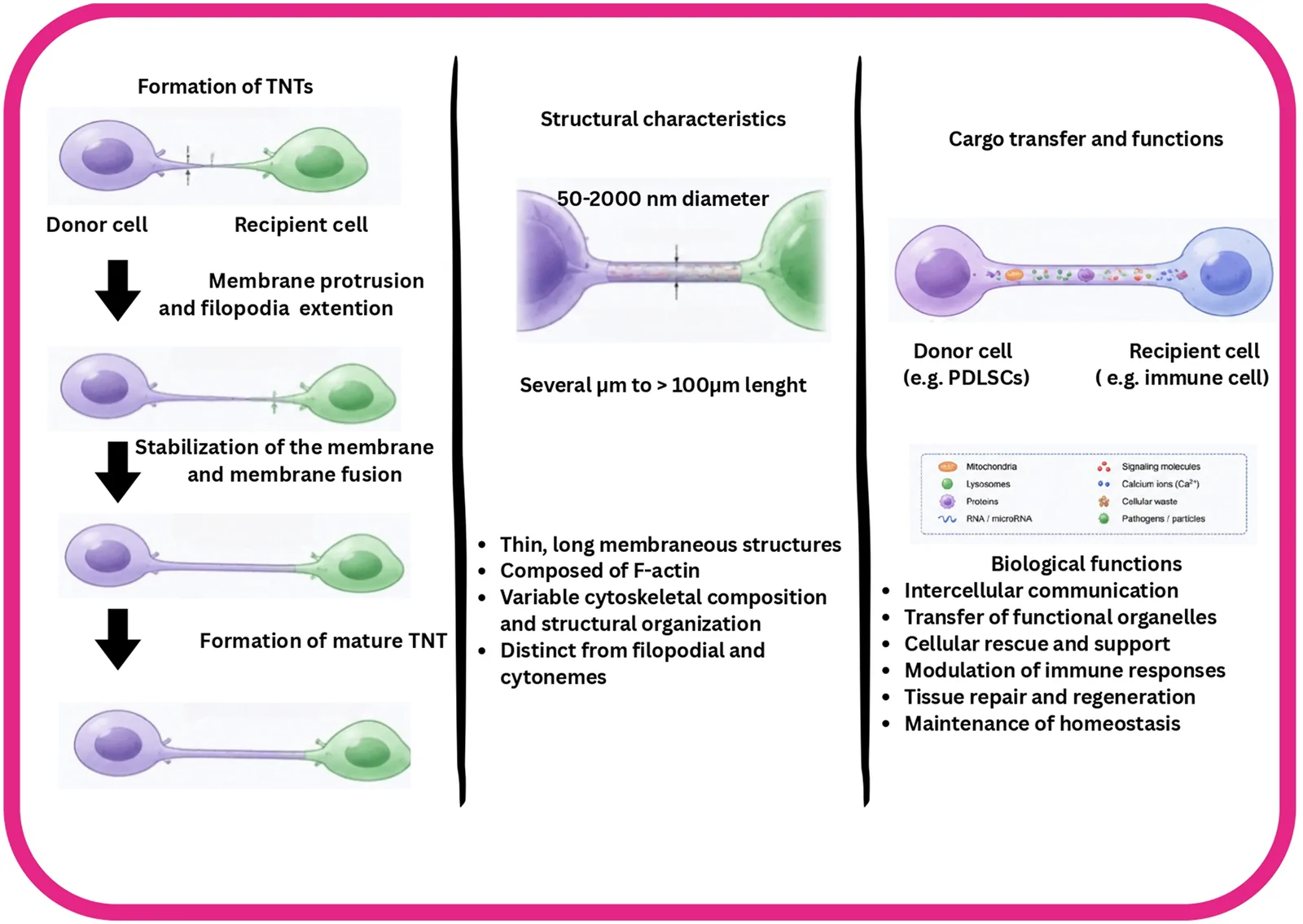
Biology of tunneling nanotubes (TNTs): formation, structure, cargo transfer, and functions. Left: TNT formation through membrane protrusion, stabilization, and establishment of a connection between donor and recipient cells. Middle: Structural characteristics of TNTs, including thin, F-actin-based membranous bridges approximately 50–2000nm in diameter and ranging from several micrometers to over 100 μm in length. TNTs exhibit structural heterogeneity, and their cytoskeletal composition and association with gap-junction proteins, including connexins, may vary depending on cell type and TNT subtype. They are distinct from filopodia and cytonemes. Right: TNT-mediated intercellular transfer of cellular cargo between cells, such as periodontal ligament stem cells (PDLSCs) and immune cells. Transferred cargo may include organelles, proteins, RNA/microRNA, calcium ions, signaling molecules, pathogens, and vesicles. These structures may contribute to intercellular communication, immune regulation, cellular rescue, tissue repair, regeneration, and homeostasis.

### 2.2 Mechanisms of TNT formation

The precise mechanisms underlying tunneling nanotube (TNT) formation remain incompletely understood; however, two major models have been widely proposed: actin-driven protrusion and cell dislodgement. In the actin-driven protrusion model, one cell extends an F-actin-rich membrane protrusion toward a neighboring cell, where membrane fusion establishes a continuous cytoplasmic bridge, enabling direct intercellular communication. The cell dislodgement model proposes that TNTs form when two cells initially establish close membrane contact and subsequently migrate apart, leaving behind a thin membranous bridge that matures into an actin-supported TNT (11, 15). TNT formation is actively regulated by coordinated remodeling of the actin cytoskeleton and several molecular mediators, including tumor necrosis factor alpha-induced protein 2 (M-Sec/TNFAIP2), leukocyte-specific transcript 1 (LST1), Myosin X (Myo10), tumor protein p53, cell division cycle 42 (CDC42) and Ras-related C3 botulinum toxin substrate 1 (Rac1), which contribute to membrane protrusion, elongation and stabilization (16–19). In addition, cellular stressors such as oxidative stress, hypoxia, inflammation, infection and mitochondrial dysfunction promote TNT formation, suggesting that TNTs represent an adaptive response that enhances intercellular communication during cellular stress, immune activation and tissue repair (20). Overall, the mechanism of TNT biogenesis appears to be influenced by both intracellular signaling pathways and the surrounding microenvironment, with the predominant pathway varying according to cell type (13).

### 2.3 Cargo transport through TNTs

Tunneling nanotubes (TNTs) function as dynamic intercellular conduits that facilitate the transfer of diverse cellular cargo between connected cells, including membrane-bound organelles, proteins, nucleic acids, vesicles, and signaling molecules, thereby influencing the physiological and functional state of recipient cells. Among these cargoes, mitochondria are the most extensively studied because of their role in maintaining cellular bioenergetics and survival under stress conditions. TNTs also mediate the transfer of lysosomes, proteins, messenger RNA (mRNA), microRNAs (miRNAs), calcium ions (Ca^2+^), and other signaling molecules, contributing to cellular homeostasis, immune regulation, and intercellular signaling (13). Collectively, the diverse cargo transported through TNTs underscores their importance in cellular rescue, tissue homeostasis, immune modulation, tissue repair and regeneration, providing a biological basis for their emerging role in stem cell biology and osteoimmunity (11, 13, 15).

## 3 TNTs in stem cells and immune cells

### 3.1 Tunneling nanotubes in mesenchymal stem cells

Mesenchymal stem cells (MSCs) utilize tunneling nanotubes (TNTs) for intercellular transfer of mitochondria, proteins, RNA, and signaling molecules, particularly under cellular stress and injury (15). Mitochondrial transfer has received considerable attention because it can improve recipient-cell bioenergetics, reduce oxidative stress, and promote cell survival and tissue repair (15). TNT-mediated communication may also influence MSC proliferation, differentiation, and immunomodulatory activity (13, 19). However, these outcomes depend on the donor and recipient cell types and experimental conditions, and the relative contributions of TNT-mediated transfer and other intercellular communication pathways require careful distinction.

### 3.2 TNTs in immune cells

TNTs have been reported in several immune cell populations, including macrophages, dendritic cells, natural killer cells, and T lymphocytes, where they facilitate the transfer of cellular cargo and signaling molecules (14, 19). Their reported functions include intercellular signaling, immune coordination, and the exchange of organelles that may influence cellular metabolism and function. However, the biological consequences of TNT-mediated communication are context-dependent and may be either beneficial or detrimental to the recipient cell. These findings provide a rationale for investigating whether TNTs contribute to communication between periodontal ligament stem cells (PDLSCs) and immune cells during periodontal inflammation and regeneration. Direct evidence establishing the contribution of TNTs to PDLSC–immune cell interactions remains an important research gap.

## 4 PDLSCs and osteoimmune regulation

### 4.1 Biological properties of PDLSCs

Periodontal ligament stem cells (PDLSCs) are multipotent mesenchymal stem cells with self-renewal and multilineage differentiation potential, contributing to the regeneration of periodontal supporting tissues (7). Beyond their regenerative capacity, PDLSCs exert immunomodulatory effects through cytokines, growth factors, extracellular vesicles, and interactions with immune cells (8–10). These properties position PDLSCs at the interface of periodontal tissue repair and immune regulation.

### 4.2 PDLSC–immune cell communication

PDLSCs interact with innate and adaptive immune cells through soluble mediators, extracellular vesicles, and direct cell-to-cell contact, influencing macrophage polarization, T-cell responses, and osteoclast activity (9, 10). Under physiological conditions, these interactions may support tissue homeostasis and repair, whereas dysregulated communication during periodontitis may contribute to persistent inflammation and alveolar bone loss (2, 4, 5).

Although these established pathways explain important aspects of PDLSC-mediated immunoregulation, they do not fully characterize the potential contribution of direct, contact-dependent intercellular cargo transfer. TNTs represent a candidate mechanism through which PDLSCs and immune cells could exchange organelles and signaling molecules. Nevertheless, findings from other MSC and immune cell models cannot automatically be extrapolated to periodontal tissues. Future studies should determine whether PDLSC–immune cell TNTs form under inflammatory conditions, identify the cargo transferred, and establish whether this transfer promotes resolution of inflammation or contributes to tissue destruction.

## 5 TNT-mediated communication in the periodontal osteoimmune microenvironment

### 5.1 Current evidence

#### 5.1.1 Current evidence

Direct evidence establishing tunneling nanotube (TNT)-mediated communication between periodontal ligament stem cells (PDLSCs) and immune cells is currently lacking. However, studies in mesenchymal stem cell (MSC) models have demonstrated mitochondrial transfer associated with changes in immune-cell function, including enhanced macrophage phagocytosis under specific experimental conditions (20–22). These findings suggest that intercellular organelle transfer may influence immune-cell metabolism and function. Nevertheless, the mechanism of transfer must be established in each experimental system, because mitochondrial transfer alone does not demonstrate that structural TNTs mediate the process.

TNT formation and intercellular organelle trafficking may also vary with the activation state and functional characteristics of the participating cells (22). Such findings highlight the importance of the cellular microenvironment in determining the formation and consequences of TNT-associated communication. Because PDLSCs possess immunomodulatory properties and interact with macrophages, T cells, and dendritic cells during periodontal inflammation, investigating whether similar communication occurs within periodontal tissues represents a relevant research direction (8–10). However, findings from other MSC and immune-cell models cannot automatically be extrapolated to PDLSCs or the periodontal microenvironment. Direct studies are required to establish whether TNTs form between PDLSCs and immune cells, identify the cargo transferred, and determine the functional consequences of these interactions under physiological and inflammatory conditions.

### 5.2 Proposed role of TNTs in osteoimmunity and periodontal regeneration

Direct evidence establishing tunneling nanotube (TNT)-mediated communication between periodontal ligament stem cells (PDLSCs) and immune cells within periodontal tissues is currently lacking. Nevertheless, studies in bone biology, stem cell research, and immunology provide a basis for investigating the potential involvement of TNT-associated communication in periodontal osteoimmunity. In skeletal systems, TNT formation has been implicated in the regulation of osteoclastogenesis (23). TNT-mediated communication has also been investigated between endothelial progenitor cells and osteoclast precursors (24). Separately, studies of mitochondrial transfer and metabolic remodeling have highlighted the importance of intercellular mitochondrial exchange in determining cellular function and fate (25, 26). These findings establish the relevance of TNT-associated communication and mitochondrial transfer to bone-related research, but they do not establish that TNT formation invariably suppresses osteoclastogenesis or promotes bone preservation (Figure 2).

**Figure 2:**
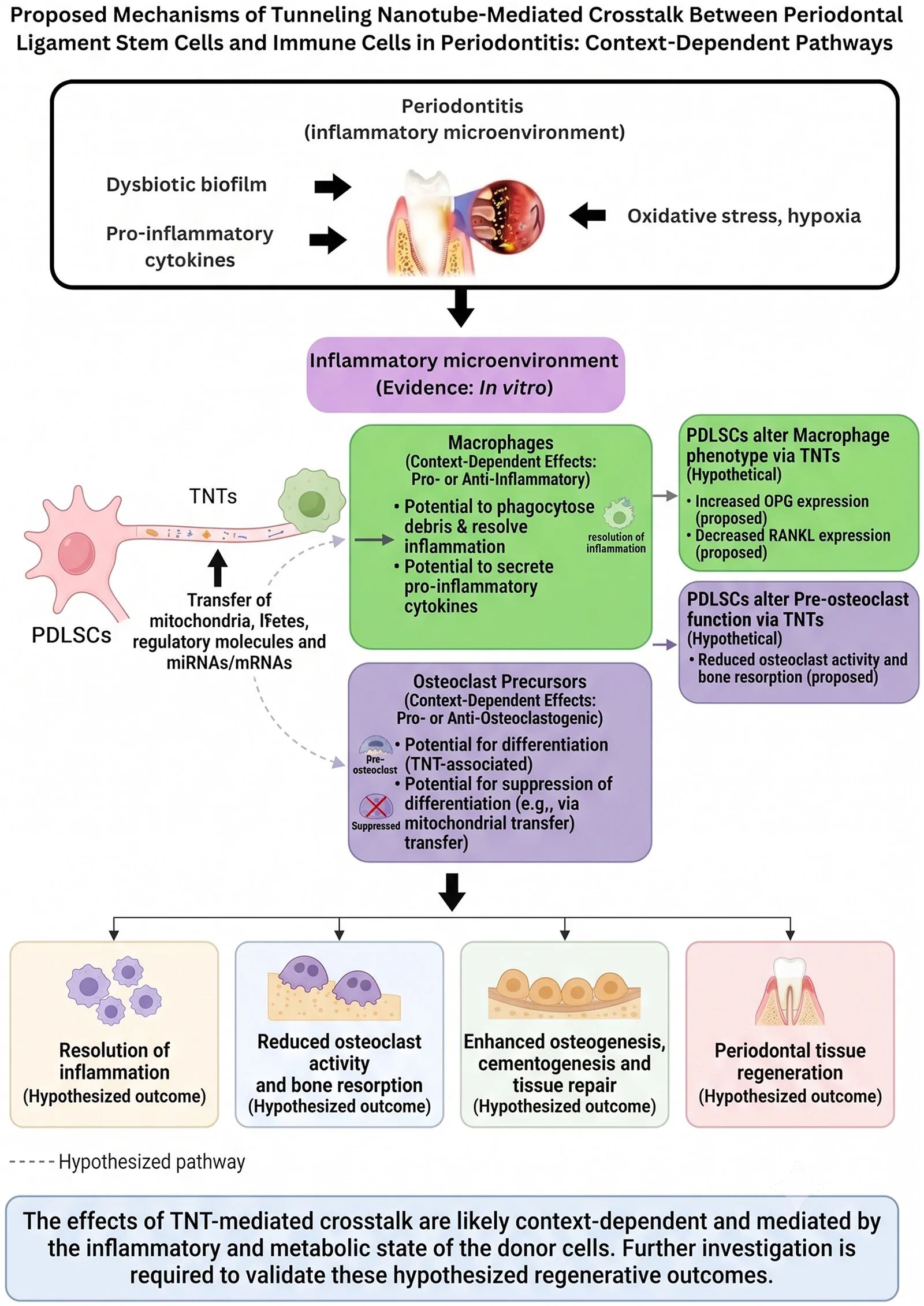
Proposed mechanisms of tunneling nanotube (TNT)-mediated crosstalk between periodontal ligament stem cells (PDLSCs) and immune cells in periodontitis. The inflammatory microenvironment, characterized by dysbiotic biofilm, pro-inflammatory cytokines, oxidative stress, and hypoxia, may influence TNT-mediated intercellular communication. *In vitro* evidence supports the presence of inflammatory responses under these conditions; however, the downstream effects of TNT-mediated transfer on macrophage polarization, osteoclast precursor differentiation, and periodontal regeneration remain hypothetical in periodontal models. Potential transfer of mitochondria, lipids, regulatory molecules, and microRNAs may exert context-dependent effects on macrophage function and osteoclastogenesis. Proposed outcomes include resolution of inflammation, reduced osteoclast activity and bone resorption, enhanced osteogenesis and cementogenesis and periodontal tissue repair. Solid arrows indicate the proposed sequence of events, whereas dashed arrows indicate hypothesized pathways. These mechanisms and regenerative outcomes require further experimental validation in periodontal models.

Evidence from other experimental systems further indicates that MSC-mediated mitochondrial transfer can influence macrophage function and cellular responses (20–22). However, the biological consequences of mitochondrial transfer may vary according to the donor cell's inflammatory and metabolic state, the functional condition of the recipient cell, and the characteristics of the transferred cargo. Wang et al, recent 2026 investigation has specifically reported mitochondrial transfer from PDLSCs to macrophages (27). Crucially, however, the observation of mitochondrial trafficking must not be presented as definitive proof of TNT-mediated transfer, as cargo exchange can likewise occur via alternative pathways such as extracellular vesicles, microvesicles, or transient membrane fusions.

Based on these intersecting lines of evidence, it is hypothesized that PDLSCs may establish direct or indirect connections with macrophages and osteoclast precursors within the periodontal microenvironment. As modeled in Figure 2, such crosstalk could theoretically enable the transfer of mitochondria and regulatory molecules, promote macrophage polarization toward an anti-inflammatory phenotype, modulate the RANKL/OPG axis, suppress osteoclastogenesis, and support periodontal tissue regeneration (4–6, 13, 20–27). Although the specific execution of these events via structural TNT conduits remains a working hypothesis requiring rigorous *in vivo* validation, these proposed pathways offer a biologically plausible framework for future therapeutic exploration in periodontal regeneration.

## 6 Therapeutic implications and future perspectives

Tunneling nanotubes (TNTs) are a potential research direction for investigating intercellular communication in periodontal inflammation and bone remodeling. Studies in other cellular systems have reported TNT-associated cargo transfer involving cellular metabolism, immune-cell function, and organelle trafficking (20–22, 25, 28–31). However, direct evidence of TNT-mediated communication between periodontal ligament stem cells (PDLSCs) and immune cells in periodontal tissues remains limited. The effects of TNT formation and mitochondrial transfer may depend on the participating cell types, transferred cargo, and local inflammatory conditions. Therefore, their contribution to periodontal inflammation and regeneration remains uncertain.

Future studies should investigate TNT formation and functional cargo transfer between PDLSCs and immune cells using live-cell imaging, three-dimensional culture systems, and experimental models of periodontal inflammation. Appropriate methods are also needed to distinguish TNT-mediated transfer from other forms of intercellular communication. Further research should examine whether these interactions influence macrophage responses, osteoclast differentiation, bone remodeling, and periodontal tissue repair.

Although TNT-mediated mitochondrial transfer has been investigated in other cellular systems, its therapeutic relevance to periodontal regeneration remains hypothetical. Whether TNT modulation can improve PDLSC function, influence osteoimmune responses, or support periodontal tissue repair requires experimental validation. Further characterization of TNTs in periodontal tissues may help determine their biological significance and whether they warrant investigation as a potential component of regenerative strategies.

## 7 Conclusion

Tunneling nanotubes (TNTs) are specialized structures involved in intercellular communication and cellular cargo transfer in several experimental systems. Their potential contribution to communication between periodontal ligament stem cells (PDLSCs) and immune cells represents an important but insufficiently investigated area of periodontal research. Findings from stem cell, immune, and bone biology suggest that TNT-associated communication and mitochondrial transfer may influence cellular metabolism, immune-cell function, and bone remodeling. However, these effects are context-dependent and cannot be assumed to suppress osteoclastogenesis or promote periodontal regeneration. Direct evidence is required to establish whether TNTs mediate functional communication between PDLSCs and immune cells in periodontal tissues and to determine the consequences for inflammation, alveolar bone remodeling, and tissue repair. TNT modulation should therefore be regarded as a potential research direction rather than an established regenerative strategy. Future mechanistic studies will be essential to determine whether and under what conditions these pathways could have therapeutic relevance to periodontal regeneration.

## References

[1] Papapanou PN Sanz M Buduneli N Dietrich T Feres M Fine DH. Periodontitis: consensus report of workgroup 2 of the 2017 world workshop on the classification of periodontal and peri-implant diseases and conditions. J Periodontol. (2018) 89(Suppl 1):S173–82. 10.1002/JPER.17-0721 29926951

[2] Hajishengallis G. Immunomicrobial pathogenesis of periodontitis: keystones, pathobionts, and host response. Trends Immunol. (2014) 35(1):3–11. 10.1016/j.it.2013.09.001 24269668 PMC3947349

[3] Kinane DF Stathopoulou PG Papapanou PN. Periodontal diseases. Nat Rev Dis Primers. (2017) 3:17038. 10.1038/nrdp.2017.38 28805207

[4] Takayanagi H. Osteoimmunology: shared mechanisms and crosstalk between the immune and bone systems. Nat Rev Immunol. (2007) 7(4):292–304. 10.1038/nri2062 17380158

[5] Hajishengallis G Chavakis T. Local and systemic mechanisms linking periodontal disease and inflammatory comorbidities. Nat Rev Immunol. (2021) 21(7):426–40. 10.1038/s41577-020-00488-6 33510490 PMC7841384

[6] Hathaway-Schrader JD Novince CM. Maintaining homeostatic control of periodontal bone tissue. Periodontol 2000. (2021) 86(1):157–87. 10.1111/prd.12368 33690918 PMC8294452

[7] Seo BM Miura M Gronthos S Mark Bartold P Batouli S Brahim J. Investigation of multipotent postnatal stem cells from human periodontal ligament. Lancet. (2004) 364(9429):149–55. 10.1016/S0140-6736(04)16627-0 15246727

[8] Tomokiyo A Wada N Maeda H. Periodontal ligament stem cells: regenerative potency in periodontium. Stem Cells Dev. (2019) 28(15):974–85. 10.1089/scd.2019.0031 31215350

[9] Liu J Wang L Liu W Li Q Jin Z Jin Y. Immunomodulatory effects of periodontal ligament stem cells and their therapeutic potential in inflammatory diseases. Front Immunol. (2022) 13:927888.

[10] Wang JY Lee YT Chang PF Chau LY. Immunomodulatory effects of stem cells derived from human periodontal ligament. J Cell Physiol. (2009) 219(3):667–76. 10.1002/jcp.21711 19160415

[11] Rustom A Saffrich R Markovic I Walther P Gerdes HH. Nanotubular highways for intercellular organelle transport. Science. (2004) 303(5660):1007–10. 10.1126/science.1093133 14963329

[12] Gerdes HH Carvalho RN. Intercellular transfer mediated by tunneling nanotubes. Curr Opin Cell Biol. (2008) 20(4):470–5. 10.1016/j.ceb.2008.03.005 18456488

[13] Valdebenito S Lou E Baldoni J Okafo G Eugenin EA. The biology of tunneling nanotubes in normal and cancer cells: functions and therapeutic potential. Cells. (2021) 10(5):1144. 10.3390/cells10051144 34068524 PMC8151460

[14] Chauveau A Aucher A Eissmann P Vivier E Davis DM. Membrane nanotubes facilitate long-distance interactions between natural killer cells and target cells. Proc Natl Acad Sci U S A. (2010) 107(12):5545–50. 10.1073/pnas.0910074107 20212116 PMC2851811

[15] Abounit S Zurzolo C. Wiring through tunneling nanotubes–from electrical signals to organelle transfer. J Cell Sci*.* (2012) 125:1089–98. 10.1242/jcs.083279 22399801

[16] Schiller C Diakopoulos KN Rohwedder I Kremmer E Von Toerne C Ueffing M. LST1 promotes the assembly of a molecular machinery responsible for tunneling nanotube formation. J Cell Sci*.* (2013) 126:767–77. 10.1242/jcs.114033 23239025

[17] Hase K Kimura S Takatsu H Ohmae M Kawano S Kitamura H. M-sec promotes membrane nanotube formation by interacting with ral and the exocyst complex. Nat Cell Biol*.* (2009) 11:1427–32. 10.1038/ncb1990 19935652

[18] Gousset K Marzo L Commere PH Zurzolo C. Myo10 is a key regulator of TNT formation in neuronal cells. J Cell Sci*.* (2013) 126:4424–35. 10.1242/jcs.129239 23886947

[19] Dupont M Souriant S Lugo-Villarino G Maridonneau-Parini I Vérollet C. Tunneling nanotubes: intimate communication between myeloid cells. Front Immunol*.* (2018) 9:43. 29422895 10.3389/fimmu.2018.00043 PMC5788888

[20] Jackson MV Morrison TJ Doherty DF McAuley DF Matthay MA Kissenpfennig A. Mitochondrial transfer via tunneling nanotubes is an important mechanism by which mesenchymal stem cells enhance macrophage phagocytosis in the *in vitro* and *in vivo* models of ARDS. Stem Cells*.* (2016) 34(8):2210–23. 10.1002/stem.2372 27059413 PMC4982045

[21] Yang Y Ren Y Li S Zhao S Guan Q Zhang B. Tunneling nanotube-mediated stem cell immunomodulation dysfunction promotes adipose inflammation and insulin resistance in GDM. Cell Rep*.* (2025) 44(11):115087. 41187060 10.1016/j.celrep.2025.116505

[22] Abyar S Khandar AA Salehi R Abolfazl Hosseini-Yazdi S Alizadeh E Mahkam M. Macrophage polarization impacts tunneling nanotube formation and intercellular organelle trafficking. Sci Rep*.* (2019) 9:14529. 31601865 10.1038/s41598-019-50971-x PMC6787037

[23] Hikita A Yana I Wakeyama H Nakamura M Kadono Y Oshima Y. Tunneling nanotube formation is essential for the regulation of osteoclastogenesis. J Cell Biochem*.* (2013) 114(5):1238–47. 23129562 10.1002/jcb.24433

[24] Li RF Zhang W Man QW Zhao YF Zhao Y. Tunneling nanotubes mediate intercellular communication between endothelial progenitor cells and osteoclast precursors. J Mol Histol*.* (2019) 50(5):483–91. 10.1007/s10735-019-09842-y 31463584

[25] Cai W Zhang J Yu Y Ni Y Wei Y Cheng Y. Mitochondrial transfer regulates cell fate through metabolic remodeling in osteoporosis. Adv Sci (Weinh)*.* (2023) 10(9):e2204871. 10.1002/advs.202204871 36507570 PMC9896036

[26] Qiang JB Wang XL Bai XY Fan XY Shi C. Research progress of tunneling nanotubes in bone biology. Zhonghua Kou Qiang Yi Xue Za Zhi*.* (2023) 58(1):86–91. 10.3760/cma.j.cn112144-20221102-00565 36642458

[27] Wang D Li M Ma J Du G Zhou J Chen J. Mitocytosis, mitophagy, and apoptosis coordinately drive mitochondrial clearance to regulate myogenic differentiation. J Adv Res. (2026) 88:519–37. 10.1016/j.jare.2026.01.065 41605286 PMC13669943

[28] Bhattacharya S Bhattacharya S Bhattacharya D. Tunneling nanotubes: a bridge for mitochondrial transfer in cancer and stem cell biology. Stem Cell Rev Rep*.* (2023) 19(1):14–28.

[29] Liu K Ji K Guo L Wu W Lu H Shan P. Mesenchymal stem cells rescue injured endothelial cells in an *in vitro* ischemia–reperfusion model via tunneling nanotube like structure-mediated mitochondrial transfer. Microvasc Res*.* (2014) 92:10–8. 10.1016/j.mvr.2014.01.008 24486322

[30] Ventre M Netti PA. Engineering cell instructive materials to control cell fate and functions through material cues and scaffold architectures for bone and cartilage tissue engineering. ACS Appl Mater Interfaces*.* (2016) 8(24):14896–908. 10.1021/acsami.5b08658 26693600

[31] Ponnaiyan D Rughwani RR Shetty G Mahendra J. The effect of adjunctive LASER application on periodontal ligament stem cells. Front Cell Dev Biol. (2024) 11:1341628. 10.3389/fcell.2023.1341628 38283989 PMC10811063

